# Paradoxical tensions in exploiting data to implement circular economy in the textile industry

**DOI:** 10.1007/s13280-023-01865-w

**Published:** 2023-05-06

**Authors:** Päivi Luoma, Esko Penttinen, Petri Tapio, Anne Toppinen

**Affiliations:** 1 Department of Forest Sciences, University of Helsinki, Latokartanonkaari 7, 00014 Helsinki, Finland; 2 Department of Information and Service Management, Aalto University School of Business, Ekonominaukio 1, 02150 Espoo, Finland; 3 Finland Futures Research Centre, University of Turku, 20014 Turun yliopisto, Finland

**Keywords:** Circular economy, Digitalization, Paradoxical tension, Sustainability, Textile industry

## Abstract

Increasing utilization of data, enabled by digitalization, constitutes a major driver toward circular economy but is not without potential paradoxical tensions. A two-round disaggregative Delphi study and analysis of the qualitative material generated in it explored these tensions. They were found to cohere around three themes: consumer concurrence, business transparency, and technology relevance. The first theme is connected with consumers’ behavior and their perceptions as to data’s value, the transparency one involves alignment of business interests and practices with data-driven developments, and the third pertains to the actual environmental impact of digital technologies used to initiate data-driven circular economy. Business decision-making should address both the positive and the negative effects, in both the short and long term. Insight as to these tensions supports discovering how businesses can successfully utilize data in their efforts promoting circular economy within the complex reality of dynamically changing business environments.

**Supplementary Information:**

The online version contains supplementary material available at 10.1007/s13280-023-01865-w.

## Introduction

In a world where climate change and limits to natural resources are significantly defining the landscape where companies do business and create value, circular economy is a necessity and a potential contributor to sustainability (Bocken et al. 2016). This is an approach aimed at circularity of products and materials, eliminating waste, and closing material loops by such means as extended customer experience, long-life goods, product-life extension, recycling, reuse of materials, and resource efficiency (Bocken et al. 2016). One mechanism with recognized potential for enabling circular economy is digitalization, with the accompanying data (Gupta et al. 2018). Exploitation of data holds particular promise for supporting circular economy through input to the operative and strategic decision-making behind such vital business functions as product design, production planning, supply chain management, and development of the fundamental business models to be employed (Lopes de Sousa Jabbour et al. 2019). Various data-driven approaches to circular economy, among them “Industry 4.0” (Awan et al. 2021), applied by means of or depending upon the collection and analysis of data, are emerging. These have already been applied to inform new ownership models and supply chain improvements (Frishammar and Parida 2019). However, the support that data could supply to a shift toward circular economy is not without potential controversy and tensions. While data could help to extend the lifespan of products, enable reverse supply chains, and save on materials, the opportunities come with a flip side: conflicting demands and interrelated contradictory economic, environmental, and social concerns. This dual nature becomes evident when, for example, blockchain is used to verify the source of products and the actors involved (Upadhyay et al. 2021). Opportunities for value embodied in novel data sources, enhanced data analytics capabilities, and data-sharing (Del Río Castro et al. 2021) exist alongside various risks and challenges: business models losing traction, one’s competitive edge vanishing, and data privacy coming under threat (Birkel and Müller 2021). The associated controversies are exacerbated by interdependencies in value chains and in material loops that often span continents, industries, and actors with varying interests.

The likelihood of tensions among very different demands is especially topical today: the use of digitalization and data in implementing circular economy is evolving rapidly, and the related practices and rules of the game are developing accordingly. Major advances call for not only identifying opportunities and benefits but also considering potential issues and complications that accompany supporting the implementation of circular economy via data. The lens of the paradox provides us with a language to discuss the associated controversies, tensions, and competing demands per the definition of “paradoxical tensions” as “contradictory yet interrelated elements that exist simultaneously and persist over time” (Smith and Lewis 2011). The same complexities of digital paradigms in sustainability transition extend beyond circular economy also, to other topical issues, such as low-carbon practices (Sharma et al. 2022).

Although recent years have witnessed growing interest in digitalization and data related to circular economy (see, for example, Di Maria et al. 2022), most researchers consider them to be purely an opportunity for advancing circular economy. So far, however, the circular economy literature has overlooked the controversies and tensions that may arise at using data to drive circular economy, with only a few exceptions (see, for example, De Angelis 2021; Upadhyay et al. 2021). There is a recognized need for further insight as to the complexities and interlinkages that data-driven digital paradigms for sustainability entail, and for empirical studies of the topic (Schad and Bansal 2018; Carmine and De Marchi 2022).

For addressing this gap connected with the complexities of digital paradigms in sustainability transition, with specific regard to circular economy, the objective behind this paper is to contribute empirically grounded insight shedding light on the paradoxical tensions that the textile industry could face when striving to utilize data to implement circular economy. It does this by addressing the following research question: *Given the complexities surrounding data-driven digital paradigms for sustainability, what paradoxical tensions emerge in utilizing data for implementing circular economy in the textile industry?*

The focus on the textile industry stems from its vast use of numerous natural resources, which creates significant environmental impacts (Palacios-Mateo, van der Meer, and Seide, 2021). However tricky it might be, a shift toward circular economy is regarded as crucial if we are to reduce the harmful textile-related environmental impacts (Ellen MacArthur Foundation 2017). Since the industry is only beginning to implement associated practices (Saha et al. 2021), stimulating well-grounded steps toward progress in this direction is all the more crucial. In work to this end, researchers have begun looking at data as one key factor that could enable circular economy in textile value chains (Fromhold-Eisebith et al. 2021), and several data-driven developments are in progress in the field, among them a digital product passport initiative at European Union level.

We found evidence of a wide spectrum of paradoxical tensions that decision-makers face. By examining them, we strengthen understanding of the complex interplay of data-driven approaches to circular economy. Our work should also aid in developing better strategies for managing them and responding efficiently. Furthermore, our insight contributes to broader discussion of the complexities of digital paradigms in sustainability transition.

## Conceptual background

### The role of data in circular economy

Together, the phenomenon of digitalization and data’s availability in rising volumes are bringing changes to the operations and activities of organizations and societies alike. As the number of data sources grows and the quantity of data generated balloons, digitalization gains greater power to be a vital enabler of circular economy (Ranta et al. 2021). Among the data relevant for circular economy activities are details of various aspects of (product and service) life cycles and more system-oriented data (connected with value networks) that can fuel knowledge-informed advances. The value most typically cultivated via data is stimulated via the transparency, optimization, and deeper understanding (e.g., of customer needs) enabled (Chen et al. 2015). When raw data, either structured or unstructured (McCallum 2005), get processed into useful information and, further, into knowledge applied to answer important questions (Ackoff 1989), data can also support reaching corporate environmental goals (Sahoo et al. 2022). With specific regard to circular economy, the added value is to be found in allowing operations- and strategy-level decisions to factor in environmental considerations readily. Thus, business functions from product design and production planning, through supply chain management, all the way to advanced business-model development can benefit (Lopes de Sousa Jabbour et al. 2019).

Five intertwined themes with specific implications for understanding the role of data in circular economy emerge from the literature. Firstly, circular economy-related data must be available—collected and managed so that they are accessible (Rajala et al. 2018). For example, digital product identities and embedded intelligence have been proposed as ways to make these details available (Mostaghel and Chirumalla 2021). Secondly, sharing of data within value chains is needed to unlock the data’s value (Gebhardt et al. 2021). In practice, the sharing of data is complicated by not just technical hurdles but also matters of content and use, since the relevant data sources encompass sensitive business details (Braglia et al. 2021). Thirdly, data initiatives need to support better decision-making at strategic and operations level so as to create value, not only in the focal firm but elsewhere in the supply chain and society at large (Gebhardt et al. 2021). This calls for data analytics capabilities that allow organizations to interpret and utilize data in operationalizing circular economy (Kristoffersen et al. 2021). Fourthly, realizing circular economy connects with new business models that could be supported by data, such as product–service systems (Lüdeke-Freund et al. 2019). Finally, solid use of data should support circular economy’s positive environment impact.

### The complex nature of textiles’ circular economy

Through their unchecked use of land and water resources, other natural resources, and chemicals, the value chains related to textiles, which extend to every part of the world, have substantial environmental impacts but also economic and social ones (Niinimäki et al. 2020). Complicating the picture still further are the industry’s expanding design and material variety, typically unpredictable nature (related in part to high levels of impulse purchasing by consumers), and short product-life cycles (Islam et al. 2021). While circular economy efforts, in all industries, are focused especially on environmental benefits, they involve addressing multiple environmental, economic, and social goals and desired outcomes simultaneously in very different temporal and spatial arenas (Sehnem et al. 2019).

In the context of circular economy, a “business performance requires growth” rationale must be balanced against the awareness that natural resources are limited (Perey et al. 2018). Hence, conflicts are bound to arise. Aiming for a transition toward circularity puts today’s businesses on a collision course, between linear and circular business models, such that fault lines may emerge within individual companies (Chizaryfard et al. 2021) and beyond. Furthermore, practices aimed at circularity may be mutually contradictory: for instance, some practices geared for reducing consumption might be confounded by efforts to extend products’ service life (whether in the same form or via new uses for the materials) or vice versa (Sehnem et al. 2019), and both may run counter to established profit models.

Many further factors could exacerbate the conflicts of legitimacy in particular contexts, connected with adoption of particular technologies, differences in organization structures, and regulatory developments—all of which may differ within and between companies, industries, or countries (Chizaryfard et al. 2021). Power structures can play a vital role in developments; for instance, global value chains controlled by a multinational corporation could well leave a smaller company in the structure little room for autonomous input on the system’s path toward circular economy (Schroeder et al. 2018). Actors in various domains might be out of step with regard to other relevant priorities too: a society’s norms and values (e.g., focusing on sustainability as ethical) may not match real-world consumer behavior (e.g., dynamics of unsustainable consumption) (Chizaryfard et al. 2021).

### The paradoxical lens

When aiming to utilize digital paradigms for implementing circular economy or any other aspect of sustainability transition, decision-makers operate in complex and dynamic business environments with competing demands and conflicting yet interrelated economic, environmental, and social concerns (Smith and Lewis 2011; Hahn et al. 2014). In a world that is growing increasingly global, complex, and contradiction-ridden, paradoxical tensions are likely to spread and intensify (González-González et al. 2019). Incorporating an approach that zeroes in on paradoxical tensions can be of particular value in managing wicked problems with complicated knots of interconnections (Schad and Bansal 2018), a class of problem often encountered with sustainability transition (Hahn et al. 2014). In the case of sustainability, paradoxical tensions are not limited to the level of individuals and organizations; systems’ inherent tensions that do not respect organizational boundaries emerge too (Schad and Bansal 2018).

Considering paradoxical tensions provides richness to the otherwise simplistic framing of competing alternatives that present clear advantages and disadvantages (Smith and Lewis 2011; Hahn et al. 2014). Whereas dilemmas are either…or problems that allow selecting one alternative over another, paradoxes necessitate exploring how several options can be pursued simultaneously (Van der Byl and Slawinski 2015). Recognizing a tension as paradoxical creates opportunities to consider the complexity of the sustainability problems and finding synergies that reconcile the tension’s poles (Schad et al. 2016; Carmine and De Marchi 2022). Though there is still some lack of clarity as to the meaning of “paradox,” the concept has been successfully used for detection of paradoxical tensions in various sustainability domains, thus enriching scholarly understanding of how individuals and organizations accept sustainability tensions, manage these, and integrate them into the evolving landscape (Carmine and De Marchi 2022). Consciously confronting tensions and reflexively tackling them aids in developing capabilities to manage them, thereby resulting in better coping strategies and efficient response (Hahn et al. 2014; Schad and Bansal 2018; González-González et al. 2019).

**Fig. 1:**
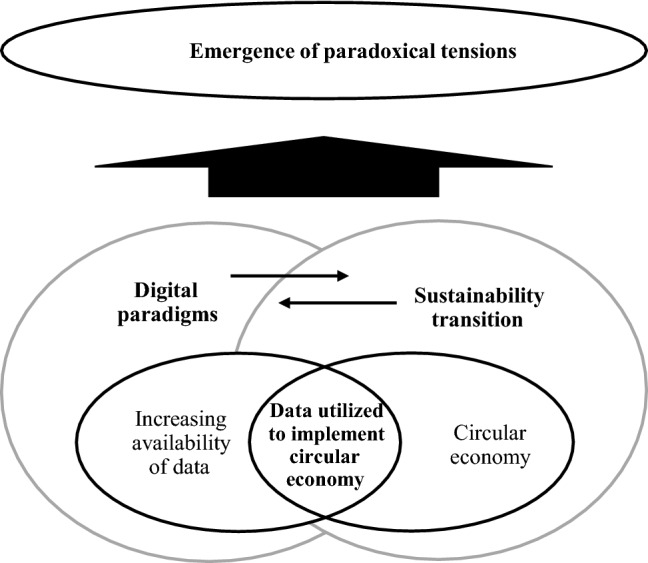
The conceptual framework of the study

Recognizing certain tensions related to utilizing data to implement circular economy as paradoxical challenges the straightforward thinking that the valuable insight and knowledge created out of data must—through better decisions by business and consumers alike—lead to reduced resource use with positive environmental impacts, the ultimate goal for circular economy. This lens forces us to consider, instead, the complex reality of sustainability problems at individuals, organization, and/or systems levels and reckon with unclear outcomes. Figure 1 depicts the corresponding conceptual framework of our study.

Academic research has successfully applied the paradox approach in work on corporate sustainability (see, for example,Hahn et al. 2018; Ivory and Brooks 2018; Carmine and De Marchi 2022). The approach is regarded as attuned well to producing a holistic view and understanding of interconnections, along with solid ability to interpret the controversial demands and diverse pressures that accompany moving toward sustainably conducted business (Hahn et al. 2014; Van der Byl and Slawinski 2015). However, the work on paradoxical tensions has been mostly conceptual, with empirical research into paradoxical tensions being viewed as challenging, especially in the sustainability context, where companies’ activities are interwoven with complex environments (Schad and Bansal 2018).

Though largely theory-oriented, a body of research on paradoxical tensions is emerging in the context of circular economy. Daddi et al. (2019) have studied the tensions between pursuing circularity goals, such as use of recycled materials, along with product competitiveness and strategies to manage said tensions. Morales (2020) has identified paradoxical tensions, such as resistance from the value chain and matching supply with demand, in circular business models. With specific focus on business models, De Angelis (2021) too has pinpointed paradoxical tensions: short-term profitability vs. long-term prosperity, competition vs. collaboration in innovation for circularity, etc. Other work, by Chizaryfard et al. (2021), has studied structural tensions in industrial progress toward circular economy. Also, studies of digital technologies’ ambiguous impacts on sustainability (Bohnsack et al. 2022; Di Maria et al. 2022) and on circular economy are emerging (Upadhyay et al. 2021), but these have not made use of the lens of paradoxes thus far.

## MATERIALS AND METHODS

Several qualitative and quantitative methods have found use in paradox studies (Andriopoulos and Lewis 2009). We chose to conduct a disaggregative Delphi study (Tapio 2003; Steinert 2009) and qualitative analysis of its results, to probe whether paradoxical tensions can be empirically observed and, secondly, identify what those paradoxical tensions might be. Hence, no a priori stance to the existence of any such tensions was taken; rather, we were eager to know whether the complexities associated with digitalization, data, and sustainability constitute fertile ground for paradoxes’ emergence. The empirical approach enabled proceeding from the observations made in the empirical setting to seek the hypotheses best explaining our observations. The qualitative approach helped to illuminate the emerging and rapidly developing field of data and circular economy beyond what is currently theorized and aided in conceptualizing potential tensions not yet recognized. As a non-consensus-oriented method that builds on experts’ views of the probable and the preferred future (Linstone and Turoff 1975; Rowe and Wright 2001), disaggregative Delphi offers a way of systematically assessing future developments and tensions by rendering experts’ arguments on future developments visible (Bell and Mau 1971; Gausemeier et al. 1998). This technique provided us with an extensive and rich body of material for answering the research question about the paradoxical tensions emerging in utilizing data for implementing circular economy in the textile industry.

**Fig. 2:**
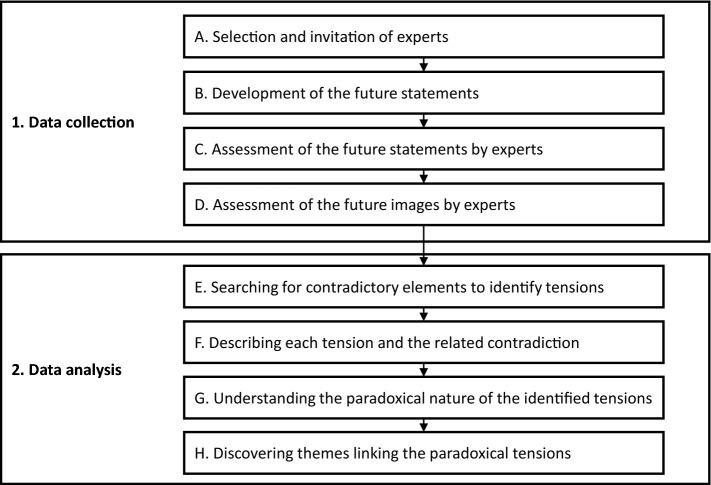
The steps of the research process

In the process we followed, invited experts assessed a set of statements about the future role of data in textiles’ circular economy and, later on, three distinct “future images,” considering a spectrum of alternative futures that represent the possible role of data in circular economy for the textile industry (Bell and Mau 1971; Polak 1973). The arguments and reasoning the experts offered form the input to the analysis that followed for uncovering paradoxical tensions. Figure 2 depicts the steps of the process followed, which are described in detail below.

### Data collection

To select and invite the experts for the study (phase A), we created an expertise matrix for making sure they represented the necessary breadth of expertise. It covered the following dimensions: circular economy, digitalization, and the major portions of textile value chains (beginning-of-life, middle-of-life, and end-of-life). In total, 85 experts were invited to take part in the process, and the final sample for the first round consisted of 33 and the second round consisted of 26 experts’ responses. Of the 26 experts informing the second round, 14 were female and 12 male, and about half of the experts were 30–44 years old or younger. This international panel represented 10 nationalities, centered mainly in Europe and North America. Table 1 presents the Delphi panelists’ organization types and areas of expertise.

**Table 1:** The second-round Delphi experts’ organization types and areas of expertise

Substance expertise^a^	Both circular economy and textiles	10
	Circular economy and also data and digitalization	6
	Circular economy, textiles, and data and digitalization	4
	Circular economy	3
	Textiles	3
Organization type^a^	Industry entity	6
	Research institution	10
	Organization involved with both industry and research	9
	Public authority	1
In total		26

^a^As reported by the experts themselves. The experts were free to identify several organization types and/or areas of expertise if relevant

A set of hypothetical statements about the future of utilizing data to support textiles’ circular economy was created. By means of a review of literature on the interface of data, digitalization, and circular economy alongside publications specifically about circular economy in the textile field (phase B), we identified five themes with related dimensions as significant for assessment of the future, and the hypothetical statements were articulated to cover these. To guarantee relevance, coverage, and a reasonable number of items, statements’ formulation was tuned in line with feedback from two of the textile industry’s circular economy experts (see Rowe and Wright 2001). The first round included 17 closed statements with Likert scale response options, each accompanied by an open-comments box for qualitative arguments, and three open questions. Supplementary Information Table S1 lists the statements presented to the experts and the literature on which they are based.

The first Delphi round, in May 2021, involved asking the experts to estimate the probable and assess the desirable development with regard to the hypothetical statements (phase C). In addition, they were asked to supply the reasoning for their answers. The approach was close to the “Argument Delphi” variant that Kuusi (1999) recommended for identifying contradictory arguments in aims of making sense of the complexity of the topic under scrutiny. For the second round, in June 2021, we created three distinct images of the future of data in circular economy for textiles, based on the first-round results (dubbed Transparency, Conflicting Interests, and Sustainable Textiles). In this Delphi round, the experts assessed the probability and desirability of the three images. Just as in the first round, they elaborated on the reasoning by supplying free-form arguments (phase D).

All in all, the qualitative material supplied in both rounds was rich and extensive, reflecting the commitment of the experts and their interest in the topic. The first-round Delphi work yielded, in all, 416 comments (coming to approx. 33 000 words) with reasoning for the replies or answering the open questions. This material was the main source for the paradoxical tensions’ identification, while the further 95 comments (3800 words) submitted as complementary input in round 2 were more like expressive reactions to the holistic images of the future.

### Data analysis

To identify potential tensions in exploiting data to implement circular economy, we began by searching for contradictory elements in the experts’ comments on the probable and preferable future developments (phase E). This entailed looking for individuals’ words and expressions indicating possible tensions, such as “on one hand … on the other hand,” “could potentially … but,” “from a different point of view,” “in the real world,” “I doubt,” and “this depends.” For example, an expert might have pointed to the importance of sharing product data to support circularity but then commented that this goes against the business interest of large retailers. In addition, we identified possibly contradictory views across the pool of experts. For example, some experts argued that personalized garments have greater “emotional durability” and, thereby, a longer life, whereas others cited studies attesting that these items are not used any longer than normal ones.

For making the tensions tangible, we created brief descriptions of them to concretize the specific contradiction manifested in each tension (phase F). By returning to the comments and arguments of the experts, we made sure to capture the key contradiction in each tension and, in addition, verified that the tensions dovetailed with notions articulated by at least two respondents. Allowing the tensions to unfold via experts’ own comments meant that we were sensitive both to those directly linked to one specific statement about the future (such as the one on embedded intelligence or personalization of textiles) and to tensions rooted in several statements (such as the tension surrounding openness of products’ life-cycle data, which was discussed in relation to not only availability of open data but also traceability and digital product passports).

To understand the paradoxical nature of the tensions identified, we then scrutinized them against the backdrop of the characteristics of paradoxical tensions. Thus, we checked that each featured an interdependent and simultaneous nature (phase G). We made sure also that they expressed a clear link to both circular economy and data, and we refined their description accordingly. This further definition distilled the set of paradoxical tensions considered to only the most central ones. In the end, nine distinct tensions of a paradoxical nature were identified, linked to 10 out of the 17 original statements assessed by the experts.

We then looked for common denominators across the nine paradoxical tensions, to understand their mutual relations (phase H). A connection to three themes—namely, consumer behavior and perceptions, business transparency and mindset, and the environmental impact of the digital technologies—was clearly visible. In this phase, we also reflected on how the themes and paradoxical tensions identified appear in the literature, to enhance understanding. The descriptions below enrich the picture via selected illustrative extracts from the experts’ responses.

## RESULTS

The results indicate that multiple tensions of a paradoxical nature accompany utilizing data to implement circular economy for textiles. Some of them are visible directly from the responses of individual experts (for example, where an expert mentions the difficulty of assessing the real-world outcome of a specific given future development), and others come to the fore only when responses of individual experts are viewed side by side (for instance, in cases of assumptions or arguments that could raise controversy among the experts). All in all, the experts’ input underlines the complexity of implementing circular economy in global textile value chains that exhibit numerous production and consumption patterns running counter to circular economy; this complexity is further increased by the use of digitization and data.

The paradoxical tensions found cohere around three themes, which we identified as consumer concurrence, business transparency, and technology relevance. Four of the tensions identified are connected with consumer behavior and perceptions by consumers—namely, whether consumers act on sustainability data, what value they see in sharing data during products’ use, and how new ownership models and personalized textiles (both benefiting from increasing availability of data) change consumer behavior. Three of the paradoxical tensions pinpointed are related to business transparency: whether common data-connected practices and openness of textiles’ life-cycle data strengthens business interests and how easily data-driven circular economy is aligned with the current business practices and processes. The last two, connected with technology relevance, involve the actual environmental impact of the digital technologies used to initiate transition toward data-driven circular economy. Table 2 presents the paradoxical tensions and the following subsections describe each of the paradoxical tensions in detail.

**Table 2:** Paradoxical tensions in utilizing data to implement circular economy

Paradoxical tension	Description of the contradictory yet interrelated elements falling under the theme
**Theme 1: consumer concurrence**	
P1.1. Consumers claim to value sustainability but do not act on sustainability information	Consumers ascribe increasing value to sustainability, but price-sensitivity rather than sustainability information guides their consumption choices
P1.2. While consumers may benefit from sharing data on textiles’ use, they may find this too intimate	Consumers can obtain social benefits by sharing data on the use of textiles, but data on consumers’ use of textiles may be too intimate or valuable to be shared
P1.3. New ownership models could either reinforce or weaken responsibility	New ownership models encourage reduced material consumption and closed loops, in line with companies’ greater interest in textiles’ life-cycle optimization, but, by not owning the textiles, consumers become detached from them and end up caring less
P1.4. Personalization can support attachment without necessarily reducing consumption	Personalization supports attachment to textiles and extends their life, reducing total consumption of textile products, but personalized textiles do not necessarily remain in use longer and are hard to resell, creating extra waste
**Theme 2: business transparency**	
P2.1. Uniform data-related practices could benefit all but may conflict with specific interests	Common data standards are needed, to guarantee interoperability related to circular economy data for textiles, but extensive variation in textile industry actors’ preparedness and interests could render global standards impossible
P2.2. Openness of life-cycle data supports companies’ sustainability story but could endanger the competitive edge	Open sharing of textiles’ life-cycle data supports the credibility of textile companies and their sustainability story but also uncovers hidden data and calls established textile industry practices into question
P2.3. Companies experience pressure for change but may be locked into business as usual	External and internal pressure to shift toward circular economy and increased transparency can drive textile companies to change, but textile companies are locked into existing practices and lack some of the capabilities needed for transformation
**Theme 3: technology relevance**	
P3.1. Embedded intelligence can increase understanding but hamper recycling	Embedded intelligence adds to knowledge of the customers and their use of textiles, but it also hampers textiles’ recycling
P3.2. Distributed-ledger technology can trace life cycles but consumes energy	Distributed-ledger technology aids in tracing textiles and their materials throughout their life cycle but also has heavy energy demands

### Theme 1: Consumer concurrence

#### P1.1: Consumers claim to value sustainability but do not act on the sustainability data

Increasingly, consumers seem to value environmental sustainability, at least in specific markets and market segments. However, the experts would like to see consumers not only become more aware of the environmental and social impact of textiles but also act on the information. In addition to noting considerable differences in consumers around the world, the experts recognized that consumers are extremely price-sensitive in their consumption choices. As respondent AR, a business, academic, and public-authority expert on circular economy and textiles, noted, “Many consumers globally are price-sensitive by necessity, don’t know about circularity, and have more immediate pressing issues to deal with.” Many stated that the environmental costs of textiles should be internalized if one wishes to guarantee genuine change in production and consumption patterns. All in all, the experts perceived it as highly challenging to communicate with consumers about sustainability, and there is a large amount of confusion as to what makes textiles sustainable.

#### P1.2: While consumers may benefit from sharing data on textiles’ use, they might find this too intimate

The experts regarded sharing of data connected with products’ use as able to bring social benefits for consumers and grant them insight as to their personal behavior and its impact. Simultaneously, it could inform fuller understanding of materials’ durability and of style preferences. The experts concluded that consumers are likely to share data if they see this as generating value and as not requiring much effort. Respondent AC, a business expert on circular economy, articulated this sentiment thus: “If it brings consumers social [benefits] (it’s socially attractive—e.g., it’s fun and/or it’s a game) and/or economic benefits, or picks up on their other interests (e.g., interest in or willingness or commitment to protecting the environment or climate), they may be happy to share their data.” That said, some usage data might be too intimate to share. Even in general, some people view clothing as a private matter, so they might not be willing to share related data. Furthermore, not all people have such a close relationship with personal-use textiles that they would be motivated to collect or share associated data. Sharing might not yield much value for every consumer.

#### P1.3: New ownership models could either reinforce or weaken responsibility

The experts perceived a need for business models other than personal ownership of textiles, such as textile-as-a-service models, to support textiles’ circular economy: “We are going to have many, different models of consuming textiles: rental, subscription, sharing, swapping, purchasing, remaking what you own, etc.” (respondent AS, a business expert on circular economy and digitalization). These new ownership models, benefiting from data, could encourage reduced material consumption and closed loops, since companies are showing greater interest in life-cycle optimization. Overall, the panel of experts stressed that moving production and consumption of textiles toward sustainability would benefit from viewing textiles as assets rather than consumables. On the other hand, by not owning the textiles, consumers become detached from them and may take less care over them. Users may find new ownership models distracting, and some might want to retain ownership of the textiles. The experts concluded that breaking through the perceived barrier to lack of consumer ownership could prove difficult except for specific product groups (e.g., work clothes or high-quality outdoor gear).

#### P1.4: Personalization can support attachment without necessarily reducing consumption

The respondents saw personalized textiles as able to support consumers’ attachment to them, since “textiles that are customized will have greater emotional durability” (respondent AN, a business expert on circular economy, textiles, and digitalization) and enhance the items’ perceived value, thereby extending their use and lifetime: Digitalization, in combination with related data on such matters as customer preferences, already provides increasing opportunities for personalization. Personalized textiles are not necessarily used any longer than others, however, and they might be harder to resell and recover. Such textiles could, in fact, cause significant amounts of additional textile waste and reduce the environmental benefits of optimized mass production. Rather than personalization, the experts emphasized the need to reduce consumption and production and to put “a focus on sufficiency and longer-lasting design and on acceptance from customers of owning and having an emotional connection to their products” (respondent AO, a business expert on circular economy and digitalization).

### Theme 2: Business transparency

#### P2.1: Uniform data-related practices could benefit all but may conflict with specific interests

The experts perceived data standards for circular economy to be necessary for avoiding non-portable and siloed data. Data reliability is a problem if no common standards or verification of compliance is agreed upon, especially with regard to environmental impacts. With the preparation of global data standards often being a protracted process, respondents highlighted that the textile industry could agree on its own standards and proceed more swiftly, if so wishing. In any case, systems’ interoperability is seen as critical. That said, textile-related value chains are extremely fragmented, so global-level collaboration on data standards is rendered challenging. As one business and academic expert on circular economy and textiles (AM) pointed out, it “requires changes on so many levels in the system, and change is slow to happen. The textile ecosystem and value chains are extremely fragmented; sectors like collection, sorting, and recycling have been operating in silos for decades.” Markets vary greatly in their players’ readiness to discuss the topic. In addition, wide gulfs in interests between actors make it even harder to arrive at common practices.

#### P2.2: Openness of life-cycle data supports companies’ sustainability story but could endanger the competitive edge

The experts perceived open life-cycle data as able to support business interests by strengthening companies’ sustainability story and enabling differentiation in the marketplace. Openness of data is in line with the general call for greater transparency and with companies’ need to make more informed decisions about their supply chains. Making data publicly available is seen as creating pressure for improvements, and those who have a good story to tell are regarded as seizing the opportunity and giving a push to others, in turn. On the other hand, the experts perceived it as hard to convince companies in the textile industry to open their data, since they may view this as counter to their business interests, and respondents cited difficulties in agreeing about who owns data. The highly competitive textile industry is not used to open data-sharing, and lack of trust among its actors could pose a stumbling block. The aforementioned AM stressed this: “It has taken ages for brands to release their supplier lists, and this is still in ‘child shoes.’ When it comes to raw materials, there is limited traceability and transparency. I doubt if the global value chains are ready [for] this level of transparency.”

#### P2.3: Companies experience pressure for change but may be locked into business as usual

Companies in textile value chains are under increasing pressure, externally and internally, to shift toward circular economy and to support this development via increased transparency reinforced by data. The most pioneering actors are seen as having strong incentives to change their data-sharing practices so as, for example, to emphasize the origin of their raw materials (such as “plastic from the sea”). However, the experts questioned companies’ readiness for the extensive business-process transformation required for a genuine shift toward circular economy, more sustainable production and consumption of textiles, and more open use of data: “Changes that need investments, [require] work, or bring even slight inconvenience will not proceed if the mindset of the whole chain is not for it” (respondent AF, an academic expert on textiles). The shift would challenge not only current ways of working but also the power balance of the actors, and it is seen as a significant challenge because of the fragmented and price-driven nature of global textile industry value chains.

### Theme 3: Technology relevance

#### P3.1: Embedded intelligence can increase understanding but hamper recycling

The experts concluded that supporting circular economy requires a better understanding of customers and their use of textiles. Here, they saw some potential in embedded intelligence—sensors embedded in the textiles that collect and access data, such as details on usage patterns and item condition, throughout the product’s life cycle—as a tool that could add to knowledge of the customers and their use of textiles. However, the experts recognized embedded intelligence as potentially hampering recycling, in that any kind of sensor attached to textiles must be removed before recycling: “Suitable for […] certain types of products and use models but, since additional components might be difficult in recycling, should not be included just for novelty” (respondent AV, an academic expert on circular economy and textiles). In addition, embedded intelligence is likely to increase lifetime costs and prove prohibitively expensive for reasonably priced consumer products, and data privacy concerns might outweigh the possible benefits.

#### P3.2: Distributed-ledger technology can trace life cycles but consumes energy

In most countries, there are increasing demands for traceability of products and materials throughout the life cycle, and the experts regarded distributed-ledger technology (blockchain etc.) as able to support this. Ideally, the full path of the product could be verified, from the source of raw materials through the useful life and all the way to recycled material starting the life of new products. On the other hand, the experts recognized that distributed-ledger technology itself imposes extensive energy demands and leads to environmental impacts: “It's not like this technology is just based in ‘the cloud’—the environmental footprint[s] of these technologies are massive” (respondent AI, an academic expert on circular economy, digitalization, and textiles). The experts also pointed out that distributed-ledger technology might not be the best way to verify the origin of products and the accuracy of data. Other tracing and verification services exist or could emerge.

## Discussion

### Implications for theory

The paradoxical tensions identified reveal a variety of necessary considerations bundled with endeavors to utilize data in support of the implementation of circular economy. They complement the emerging discussion on the paradoxical tensions in circular economy (see, for example, Morales 2020; De Angelis 2021) by capturing tensions specific to utilization of data in this context. The paradoxical tensions identified challenge the straightforward conclusion that the use of data, and related digital technologies, will self-evidently result in reduced resource use with positive circular economy and environmental impacts. These tensions prompt us to consider the complex reality of implementing circular economy, bundled with ambiguous impacts. This conclusion is consistent with the emerging body of literature on digital technologies in relation to sustainability (Bohnsack et al. 2022). The system-inherent nature of the paradoxical tensions (Schad and Bansal 2018) influences business but also touches a much larger set of decision-makers—regulators and investors among them, along with all players connected with the textile ecosystem. They all need to understand the complexity of pursuing more sustainable production and consumption practices.

The tensions associated with the **consumer concurrence** theme draw attention to how crucial it is for consumers to be on board if the data use is going to support circular economy fully. Consumers both produce and use circular economy data, and they determine the broader acceptability and, hence, feasibility of data-driven solutions. Also, recent work emphasizes consumers’ vital role more broadly for acceptance and implementation of circular economy and of related practices and business models (Daddi et al. 2019; Agrawal et al. 2022). Guiding consumers toward more sustainable consumption behavior is crucial (Freudenreich and Schaltegger 2020; Mostaghel and Chirumalla 2021). In addition to the aforementioned possible conflicts between societal norms/values and actual behavior as exemplified by desiring sustainability vs. maintaining unsustainable consumption (Chizaryfard et al. 2021). Also, scholars have recognized the ethics implications of pushing customers to provide information on products’ use throughout a product’s lifetime (Birkel and Müller 2021).

The tensions related to new ownership models and personalized textiles draw attention to the fact that environmental benefits do not follow automatically from data-supported circular business models; rather, the outcomes depend on how these models change real-world consumption and production practices (Daddi et al. 2019; Ki et al. 2021). Although research has identified new ownership models that could support extending companies’ ownership of products over their full service life (Huynh 2021) and creating a sense of business-level responsibility for the materials (Fischer and Pascucci 2017), our findings show that actual consumption behavior could well eclipse these potential benefits. In addition, uncertainty exists as to whether the customer base on a larger scale would welcome new ways of consuming textiles and prefer more circular but potentially also more expensive solutions (Fromhold-Eisebith et al. 2021).

The paradoxical tensions related to **business transparency** demonstrate that utilizing data as a tool for establishing circular economy calls into question some existing practices of the textile industry. On the positive side are the potential benefits of collaboration, data’s sharing, and transparency; the literature specifically stresses the opportunities for collaborative innovation involving both customers and suppliers and for supply chain improvements, alongside gaining consumers’ and investors’ trust (Garcia-Muiña et al. 2018; Sodhi and Tang 2019). On the other hand, the costs of gathering the information, a lack of uniform definitions and methods, the competition landscape, and shortcomings in data availability are among the impediments. It is recognized in the literature that harnessing the value of data in a networked circular economy setting necessitates collaboration and efficient flow of information along the supply chain (Brown et al. 2019; Luoma et al. 2021), but finding balance between collaboration and competition, a recognized paradoxical tension facing companies, is a delicate matter involving both an actor’s decisions to propel circular economy implementation and its utilization of digitalization (De Angelis 2021). Utilization of data might create economic value for some actors while simultaneously decreasing the value for others (Kouhizadeh et al. 2019; Morales 2020), and new approaches might lead to some business models losing traction and a given player’s competitive edge vanishing (Birkel and Müller 2021). Another piece of the picture is regulatory pressure creating increasing incentives for businesses to implement circular economy-supporting strategies and practices, including transparency of their activities (Awan et al. 2021; Moktadir et al. 2020). This factor too will significantly influence the future landscape.

Finally, the paradoxical tensions in the **technology relevance** category point to digital technologies’ potential benefits and to evident environmental costs both. In increasing numbers, studies are highlighting that adoption of digital technologies could yield environmental, social, and economic benefits. For example, embedded intelligence could aid in monitoring and optimization of product and material flows along entire value chains and throughout life cycles (Awan et al. 2021; Ingemarsdotter, Jamsin, and Balkenende, 2020), and distributed-ledger technology could support verifying the sources of products and materials (Upadhyay et al. 2021). However, these technologies can create problems also, such as considerable consumption of energy, substantial use of finite resources, and harm to the environment (Kouhizadeh et al. 2019; Grigore et al. 2020). From perspectives not directly related to resource use, developments in technology such as new ways of product identification, and advancement in textiles’ recycling can affect the persistence of technology-related tensions. One must carefully weigh the benefits against the disadvantages, evaluating both short-term and long-term impacts, when setting out to realize circular economy by means of digital technologies (Matos et al. 2020). At the same time, we must remember that no technology is a silver bullet. This is largely because none of them exists in isolation. For instance, efforts to assess the environmental impact are complicated by digital technologies’ extensive interconnection; often, one technology cannot be adopted on its own, without others (Cagno et al. 2021).

By empirically studying paradoxical tensions in the context of textiles’ circular economy and the utilization of data, we open new research domains to paradox research involving sustainability. Our core contribution lies in using the lens of the paradox for detection purposes, as a tool for identifying and investigating the nature of tensions (Carmine and De Marchi 2022). Our project based on a disaggregative Delphi study introduces a practical way to understand and conceptualize paradoxical tensions in the emerging interdisciplinary field of sustainability shifts and digitalization.

More broadly, our work makes visible key paradoxical tensions inherent to thinking about the future. When any individual expert ponders on probable and preferred futures, the assumptions and reasoning (related to both opportunities and threats) might vary between futures; competing arguments are likely to arise; and an expert may have a non-coherent vision, featuring conflicting elements. One’s preferred image of the future might overlook some major restrictions, or a pessimistic vision deemed probable might not recognize key opportunities—thereby representing another paradoxical tension.

### Practical implications

The study pinpointed nine paradoxical tensions that business practitioners, the textile industry, and stakeholders at large should address and efficiently manage when utilizing data to implement circular economy. Insight as to these tensions supports discovering how businesses can successfully utilize data while promoting circular economy in the complex reality of dynamic business environments. Business decision-making should address both the positive and the negative effects of utilizing data in advancing the circularity transition. Crucially, factoring in economic, environmental, and social impacts in the short and long term should guarantee that the societal benefits of utilizing data exceed its costs and disadvantages.

### Limitations and further research

Though pinpointed in a particular research context, the tensions identified are not limited to textile value chains alone; we would expect our findings and approach to be valuable in other industries. That said, the picture could be broadened: while we focused on environmental sustainability related to circular economy and digitalization, the social impacts too need acknowledgment (Mukhuty et al. 2022). The results are limited to the insights and expertise of the involved experts, and thus, for example, understanding consumer behaviors/attitudes could be widened with specific expertise on these topics. Also, the strategies that businesses can apply to cope with paradoxical tensions were beyond our study’s scope.

Supporting systemic shift toward circular economy requires further research on assessing and understanding the attendant paradoxical tensions. More attention specifically to the interface with digitalization is still needed, with empirical studies being especially valuable for understanding paradoxical tensions in various business and societal contexts including understanding consumer behaviors. Studies could fruitfully discuss and conceptualize the tensions’ origin too. This is relevant with regard to all three loci of “balancing acts” we identified: consumer concurrence, business transparency, and technology relevance. In combination, studying the origins and the ways of managing the paradoxical tensions enrich knowledge of how a positive impact of digital tools may be guaranteed.

## Conclusions

Our project offers empirical examination of paradoxical tensions that the textile industry faces when striving to utilize data in pursuit of circular economy. By pinpointing nine paradoxical tensions present in utilizing data to implement circular economy, the study made visible the complex nature of digital paradigms within sustainability transition. Importantly, the paradoxical tensions were found to cohere around the three themes presented above. The first theme is connected with consumers’ behavior and their perceptions as to data’s value, the transparency one involves alignment of business interests and practices with data-driven developments, and the third pertains to the actual environmental impact of digital technologies used to initiate data-driven circular economy. Insight as to these tensions supports discovering how businesses can successfully utilize data while promoting circular economy in the complex reality of dynamic business environments. Our work should also aid in developing better strategies for managing them and responding efficiently. Furthermore, our insight contributes to broader discussion of the complexities of digital paradigms in sustainability transition.

## Supplementary Information

Below is the link to the electronic supplementary material.

Supplementary file1 (PDF 949 KB)
