# Supplementary material for: Paradoxical tensions in exploiting data to implement circular economy in the textile industry
Source: Ambio. 2023 May 6;52(8):1400–13. doi: 10.1007/s13280-023-01865-w (PMC10272033; doi:10.1007/s13280-023-01865-w)
Supplement: Supplementary file 1 — Supplementary file1 (PDF 949 KB) [file 13280_2023_1865_MOESM1_ESM.pdf]

Ambio

Electronic Supplementary Material

This supplementary material has not been peer reviewed.

Title: **Paradoxical tensions in exploiting data to implement circular economy in the textile industry**

Authors: Päivi Luoma, Esko Penttinen, Petri Tapio, Anne Toppinen

Table S1. The hypothetical statements presented to the experts and the background of the statements

Theme and dimension, with description	Statements presented to the experts	References
**AVAILABILITY OF CIRCULAR-ECONOMY DATA**		
1. **Use of digital identities:** Product life-cycle data form a prerequisite for circular economy. Digital product identities have been proposed as one way to make these details available to all actors in the value network.	Please estimate the probable/desirable share of textiles having an attached digital identity, such as a "digital biography" or "digital passport," with information on the product's life cycle in digital form in 2035.	Rajala et al., 2018
2. **Use of embedded intelligence:** Embedded intelligence enables monitoring product and material flows across value chains and throughout life cycles. Via the data generated, processes and supply chains could be optimized and controlled, for greater efficiency and value.	Please estimate the probable/desirable share of textiles containing embedded intelligence – sensors embedded in the textiles – that can collect and access data, such as details on usage patterns and item condition, throughout the product's life cycle in 2035.	Ingemarsdotter et al., 2020
3. **Textile-users' sharing of data:** Data on the use phase of textile products enable, for example, improved product design and models geared for servitized business. In addition, it could increase awareness among customers as to their use of textiles and its impact.	Please estimate the percentage of textile-users who will probably/preferably share data with the textiles' retailers and/or producers during the product's service life. An example is data on how often the product gets used.	Mostaghel & Chirumalla, 2021
4. **Traceability of textiles:** The complex and global nature of textile value chains raises the question of traceability of products and materials, including that of wood-based materials with biodiversity impacts. Data can add value by affording traceability.	Please estimate the share of wood-based textiles (as a percentage of the volume of wood-based textiles produced worldwide) for which traceability of all wood-based fibers' origin back through the value chain will be probable/desirable in 2035.	Agrawal & Pal, 2019
**SHARING OF CIRCULAR-ECONOMY DATA**		
5. **Availability of open life-cycle data:** Implementing circular economy in textile value chains renders the exchange of data among its actors important. The use of open data sources, for life-cycle data especially, could enable collaborative efforts toward circular economy.	Please estimate the share of textiles (as a percentage of the volume of textiles produced worldwide) for which it is probable/desirable that products' life-cycle data will be publicly available via open data sources and, hence, free for anyone to use or redistribute.	Brown et al., 2019
6. **Existence of global data standards:** The competitive	Please estimate the extent to which there will probably/preferably be	Niinimäki et al., 2020

	business environment of the global textile industry is likely to discourage companies from sharing data unless common standards exist. Global standards enable interoperability across actor and system boundaries.	global data standards in place for textiles' circularity-linked lifetime data (enabling data-sharing and interoperability between stakeholders) in 2035.	
7.	**Existence of European Union regulation:** Enabling regulation is needed if a level playing field and incentives for circular economy's implementation are going to be created. In the European Union, several circular-economy-related initiatives are yet to be executed.	Please estimate to what extent the European Union regulations in force in 2035 will probably/preferably require that textiles' circularity-related lifetime data be freely available.	Saha et al., 2021
8.	**Use of distributed-ledger technology:** Distributed-ledger technology (blockchain etc.) can multiply the transparency and reliability of circular-economy operations. It can assist in verifying, for example, the source of materials or products and the actors involved.	Please estimate the probable/desirable share of textiles (as a percentage of the volume of textiles produced worldwide) for which the raw materials' origin (e.g., initial location) is verified by distributed-ledger technology, such as blockchain, in 2035.	Kouhizadeh et al., 2019
**USE OF CIRCULAR-ECONOMY DATA IN BUSINESS DECISION-MAKING**			
9.	**Integration into business-management systems:** Integrating circular-economy data into business-management systems enables monitoring and analysis of such factors as the waste generated and resource-efficiency. It could support strong strategic and operations-level decision-making.	Please estimate to what extent circular-economy data will probably/preferably be integrated into business-management systems and other software in textile-related business in 2035.	Lopes de Sousa Jabbour et al., 2019
10.	**The share of structured data:** Usually, applying data analytics to extract value from data demands the availability of sufficiently structured data. However, vast volumes of today's data are generated in unstructured form.	Please estimate the probable/desirable share of structured data – data with a standard format through which they are easily accessible and exploitable – as a percentage of all data used in the context of circular economy in 2035.	McCallum, 2005
11.	**Reliability of the data:** As the role of circular-economy data in decision-making grows, reliability will be increasingly important. Exploiting data on wood-based textiles' value chains could build on the data-management systems already in place in the forest industry.	Please estimate how often issues of data reliability will probably/preferably restrict the use of data for circular economy in wood-based textile value chains in 2035.	Gupta et al., 2018
12.	**Data-ownership problems:** Contractual and ownership arrangements might limit the use of	Please estimate to what extent data-ownership issues will probably/preferably restrict data's	Brown et al., 2019

circular-economy data. The emerging value chains for wood-based textiles could introduce practices that offer transparency of data ownership.	utilization for 2035’s circular economy in wood-based textile value chains.	
**NEW CIRCULAR BUSINESS MODELS**		
13. **Existence of new ownership models:** Fresh ownership models could extend companies’ ownership of products over their full service life. This sort of data is required for value creation and for an optimal service life.	Please estimate the probable/desirable share of textiles that will be owned by the producer or retailer throughout their life cycle in 2035 (as a percentage of the volume of textiles produced worldwide).	Huynh, 2021
14. **The presence of personalized textiles:** Personalized textiles could make textiles more valuable for the customers, thus increasing their utility and lengthening their time in use. Both this personalization and the efficiency of the operations related to it require data.	Please estimate the probable/desirable share of textiles (as a percentage of the volume of textiles produced worldwide) that in 2035 will have been personalized on the basis of the user’s needs (e.g., via digital services that recommend or design products in line with user preferences).	Freudenreich & Schaltegger, 2020
**CIRCULAR ECONOMY’S IMPACTS ON THE ENVIRONMENT**		
15. **The share of recycled fibers:** Introducing recycled fibers as alternative raw materials for textiles cycles today’s waste to tomorrow’s new value. These fibers herald circularity-oriented transformation of textile value chains, including customer acceptance.	Please estimate the probable/desirable share of textiles produced from recycled fibers in 2035 (as a percentage of the volume of textiles produced worldwide).	Niinimäki et al., 2020
16. **The share of wood-based fibers:** More environment-friendly substitutes for cotton and oil-based synthetic textiles are sought. Wood-based cellulosic fibers, with more environment-friendly production technologies emerging, provide an alternative.	Please estimate the probable/desirable share of textiles produced from wood-based cellulose fibers in 2035 (as a percentage of the volume of textiles produced worldwide).	Islam et al., 2021
17. **Use of digital nudging:** Sustainable consumption choices are needed for reducing the environmental impact of textiles. Digital nudging that exploits digitalization and related data could serve as a tool to encourage circularity-focused consumption choices.	Please provide your view on how widely digital nudging (encouraging consumers toward more sustainable consumption choices) will probably/preferably be applied to guide textile-users toward sustainable consumption in 2035.	Freudenreich & Schaltegger, 2020
